# Supplementary material for: A national professional development program fills mentoring gaps for postdoctoral researchers
Source: PLoS One. 2023 Jun 14;18(6):e0275767. doi: 10.1371/journal.pone.0275767 (PMC10266628; doi:10.1371/journal.pone.0275767)
Supplement: S1 Table — (PDF) [file pone.0275767.s001.pdf]

S1 Table. Demographic information of survey respondents and national postdoc population

Variable	Category	Pre-Post Matched (*n* = 215)	Pre-Survey (*n* = 1151)	National Postdoc Population (McConnell et al., 2018)
Gender	Female	146(67.9%)	732(63.6%)	4014(53.1%)
	Male	49(22.8%)	267(23.2%)	3546(46.9%)
	Others	2(0.9%)	19(1.7%)	–
	Unknown	18(8.4%)	133(11.5%)	–
Ethnicity	White/Caucasian	90(41.9%)	525(45.6%)	4674(60.3%)
	Asian or Asian American	40(18.6%)	221(19.2%)	1924(24.8%)
	Hispanic or Latino/Latina/Latinx	25(11.6%)	100(8.7%)	513(6.6%)
	Black or African American	20(9.3%)	47(4.1%)	202(2.6%)
	Multiracial/More than one race	14(6.5%)	80(6.9%)	–
	Middle Eastern or Northern African	4(1.9%)	27(2.4%)	–
	Prefer to self-describe	0	14(1.2)	–
	Unknown	22(10.2%)	137(11.9%)	–
Discipline	Biological/Medical Sciences	120(55.8%)	619(53.8%)	5150(67.9%)
	Physical Sciences/Engineering /Computer Science	30(13.9%)	168(14.6%)	1764(23.2%)
	Humanities and Social Sciences	33(15.4%)	173(15.0%)	635(8.4%)
	Others	12(5.6%)	51(4.4%)	36(0.5%)
	Unknown	20(9.3%)	140(12.2%)	–
Country	The United States	92(42.8%)	457(39.7%)	3718(49.1%)
	Others	100(46.5%)	553(48.1%)	3851(50.9)
	Unknown	23(10.7%)	141(12.2%)	–

Note. Biological/Medical Sciences include biological and life sciences, medical sciences, agriculture and natural resource sciences, and earth, environmental, atmospheric, and ocean sciences; Physical Sciences/Engineering/Computer Sciences include computer, information, and technological sciences, engineering, mathematics and statistics, and physical sciences; Humanities/Social Sciences include Education, Humanities, Psychology, and Social, behavioral, and economic sciences.
