# Supplementary material for: A national professional development program fills mentoring gaps for postdoctoral researchers
Source: PLoS One. 2023 Jun 14;18(6):e0275767. doi: 10.1371/journal.pone.0275767 (PMC10266628; doi:10.1371/journal.pone.0275767)
Supplement: S2 Table — (PDF) [file pone.0275767.s002.pdf]

S2 Table. Brief Description of the learning activities in *Succeeding as a Postdoc*

Module	Leaning Activity	Instructions
Module 1	Identity Grid Activity	Reflect on how you think about and define yourself.
	Role Grid Activity	Reflect on the transition you are going through and list a series of generic roles that postdocs have in their professional environment.
	Community of Practice Diagram	Create a diagram displaying your current research group and working relationships that exist across your group.
	Mapping Your Goals Grid Activity	Reflect on your goals and expectations for your postdoc and develop a timeline for your postdoc and the milestones that will lead to your success.
Module 2	Informational Interview Plan Grid Activity	Plan for an informational interview.
	Putting Your Career into Action	Identify obstacles and strategies to overcome obstacles on your pathway to success.
Module 3	Resilience Action Plan	Find an action plan for developing resilience.
Module 4	Social Identity Grid Activity	Identify your social identities (gender, race, sex orientation, etc.)
