# Supplementary material for: A national professional development program fills mentoring gaps for postdoctoral researchers
Source: PLoS One. 2023 Jun 14;18(6):e0275767. doi: 10.1371/journal.pone.0275767 (PMC10266628; doi:10.1371/journal.pone.0275767)
Supplement: S3 Table — (PDF) [file pone.0275767.s003.pdf]

S5 Table. Means and standard deviations of self-reported skills on the pre- and the post-course surveys, and the follow-up repeated measures ANOVA results with the sample of postdocs only. Data were from *The Postdoc Academy: Succeeding as a Postdoc* from February 2020 through January 2022 (pre-course survey, *n* = 178, post-course survey, *n* = 178).

	Pre-Course Survey (*n* = 178)		Post-Course Survey (*n* = 178)					
Variable	*M*	*SD*	*M*	*SD*	*SS*	*df*	*F*	η^2
career transition	2.8	0.9	3.3	0.8	23.18	1	47.37***	0.06
career planning	2.8	1	3.5	0.9	38.71	1	76.59***	0.13
collaborative research	3.1	0.9	3.5	1	14.17	1	28.18***	0.04
resilience	3.3	0.8	3.8	0.8	20.32	1	51.30***	0.08
self-reflection	3.5	1	4	0.8	18.85	1	37.27***	0.07

Note. *SS* = Sum of Squares, *df* = degree of freedom; ****P* < 0.001, two-tailed.
