# Supplementary material for: A national professional development program fills mentoring gaps for postdoctoral researchers
Source: PLoS One. 2023 Jun 14;18(6):e0275767. doi: 10.1371/journal.pone.0275767 (PMC10266628; doi:10.1371/journal.pone.0275767)
Supplement: S4 Table — (PDF) [file pone.0275767.s004.pdf]

S6 Table. Pooled hierarchical regression analysis summary for gender, ethnicity, discipline and country of origin predicting skill development with the sample of postdocs only. Data were from *The Postdoc Academy: Succeeding as a Postdoc* from February 2020 through January 2022 (*n* = 178).

	Career Transition			Career Planning			Collaborative Research			Resilience			Self-Reflection		
	B	*SE(B)*	*t*	*B*	*SE(B)*	*t*	*B*	*SE(B)*	*t*	*B*	*SE(B)*	*t*	*B*	*SE(B)*	*t*
**Step 1**															
pre-survey	0.34	0.08	4.34***	0.40	0.07	5.36***	0.49	0.08	5.89***	0.45	0.08	5.89***	0.34	0.07	5.17***
R^2	0.13***			0.17***			0.2***			0.19***			0.15***		
**Step 2**															
pre-survey	0.33	0.08	4.28***	0.40	0.07	5.43***	0.49	0.08	5.88***	0.43	0.07	5.88***	0.35	0.07	5.41***
female	0.31	0.16	1.88	0.19	0.17	1.07	0.13	0.19	0.70	-0.16	0.17	-0.97	0.15	0.15	0.98
majority	-0.13	0.14	-0.91	-0.33	0.15	-2.18*	-0.18	0.17	-1.09	-0.56	0.13	-4.19***	-0.34	0.13	-2.52*
Discipline(biomedical)	0.10	0.18	0.52	0.07	0.20	0.32	-0.12	0.22	-0.55	-0.08	0.17	-0.45	0.02	0.18	0.09
Discipline(physical)	0.10	0.21	0.49	0.08	0.24	0.32	-0.09	0.25	-0.37	-0.09	0.20	-0.43	-0.09	0.21	-0.43
Country(highHDI)	-0.12	0.08	-1.51	-0.05	0.09	-0.63	0.05	0.10	0.55	0.06	0.08	0.71	-0.09	0.08	-1.15
$R^2(\Delta R^2)$	0.18(0.05)			0.22(0.05)			0.21(0.02)			0.28(0.09)**			0.22(0.07)*		
