# Supplementary figures and images for: A national professional development program fills mentoring gaps for postdoctoral researchers
Source: PLoS One. 2023 Jun 14;18(6):e0275767. doi: 10.1371/journal.pone.0275767 (PMC10266628; doi:10.1371/journal.pone.0275767)

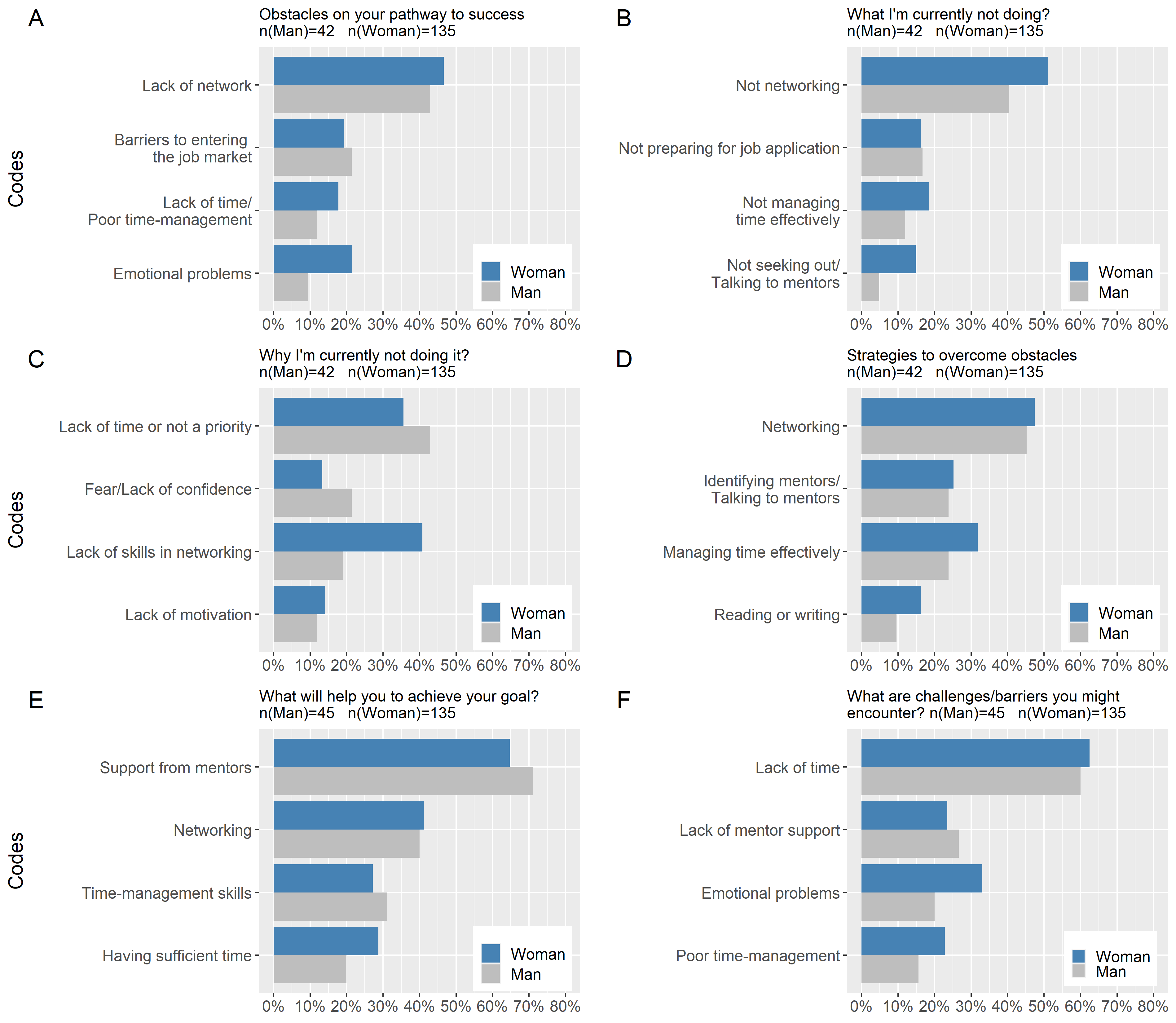

Supplement: S1 Fig — (TIF) [file pone.0275767.s006.tif]

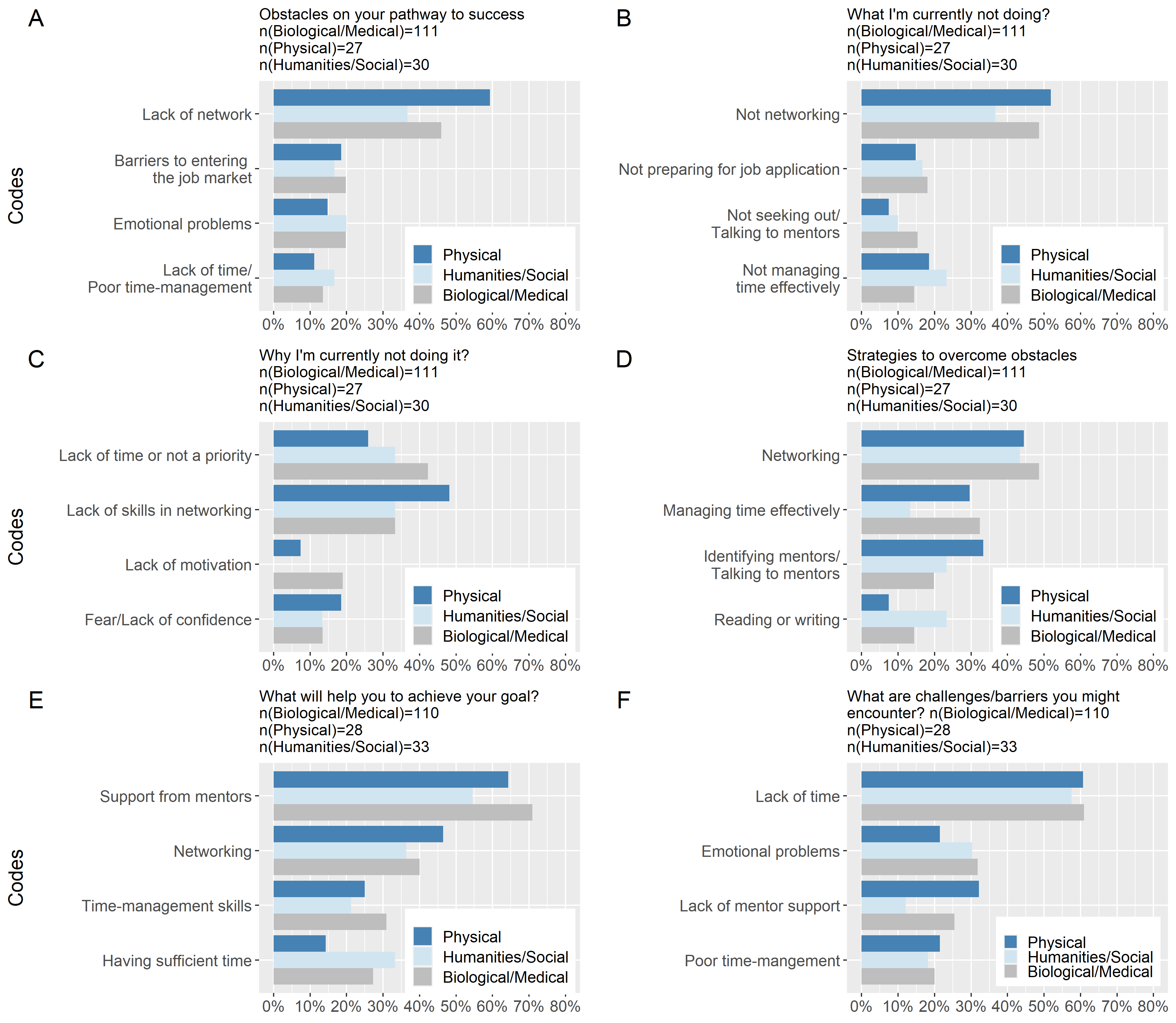

Supplement: S2 Fig — (TIF) [file pone.0275767.s007.tif]

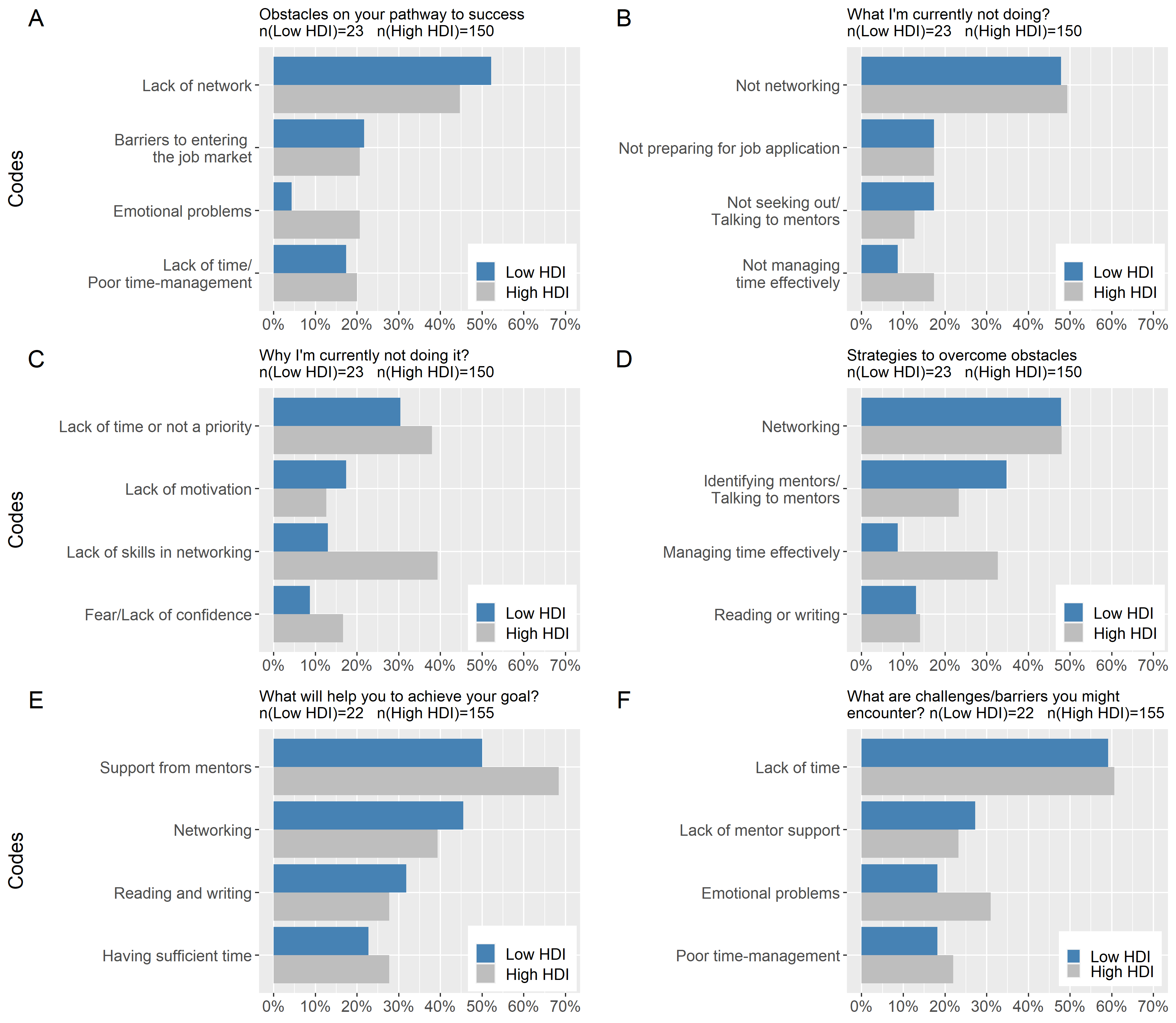

Supplement: S3 Fig — (TIF) [file pone.0275767.s008.tif]
