# Supplementary material for: A national professional development program fills mentoring gaps for postdoctoral researchers
Source: PLoS One. 2023 Jun 14;18(6):e0275767. doi: 10.1371/journal.pone.0275767 (PMC10266628; doi:10.1371/journal.pone.0275767)
Supplement: Attachment — Submitted filename: v3_ResponsesToComments.docx [file pone.0275767.s009.docx]

| **Comments** | **Responses to the comments** | **Page#** |
| --- | --- | --- |
| **Reviewer 1** |  |  |
| This manuscript presents on an evaluation of an online course for postdoctoral fellows. The team build an online module to help postdocs with their career development. The evaluation included a pre and post survey that assessed one’s self reported perception of skill improvement. Overall, the evaluation and methodology used is rigorous, as is the data analysis. They team used both qualitative and quantitative approach for this mixed method design. The data analysis included subgroup comparisons to determine if there was a different impact for those who are underrepresented, as well as gender, country, and discipline. Some minor comments may help improve the manuscript. Overall, the manuscript is extremely well-written. | Thank you for the very positive comments about the content of our project, the analyses, and kind words about the quality of the writing. |  |
| 1. The introduction seems quite lengthy. This could be shortened with a sharper focus on postdoctoral training. | The Introduction has been shortened by 249 words (4%). The section “Effects of skill building on postdoctoral success” has now been summarized into a few sentences. The revised Introduction now focuses on postdoctoral training and their professional development. | P. 5-6 |
| 2. On page 8, when discussing the sample, this seems like an extremely biased sample. That is, those who completed the course and the post survey would more likely be those who saw the potential for the module to help them. Those who dropped or didn’t complete are likely those who didn’t see the value or did not think the course was helping them. | We acknowledged that our participants comprised a self-selected and engaged subgroup, which was addressed as one of the limitations of the study. Indeed these are the individuals who opted in to take advantage of the professional development opportunity, and so more than likely have a positive inclination toward its value. We present demographic characteristics of the pre-survey sample and national postdoc population (see S1 Table) and noted a high degree of equivalence between our sample and the other two in demographic distribution, indicating at least that such self-selection doesn’t shift the demographic representation. | p.8  p. 26 |
| 3. The definition of Engaged Learner seems quite broad. Is it that it includes anyone who has done one exercise within the module? So, if someone only watched a 5 min video, they would be considered Engaged? To me, I would see someone who is engaged as someone who has completed about 50% of the work. | We agree. We changed “engaged learners” into “course learners”, referring to people who are engaged with at least one course activities. | p. 8 |
| 4. In Table 1, Matched sample is listed as a row, but not described in text. | We now added the matched sample in text, which refers to participants who responded to both the pre- and post-course surveys. | p.7 |
| 5. On page 11 in the discussion of the prompts, I think the explanation of the prompts could be a bit clearer. I was uncertain as to how they were presented to the postdoc and if they were part of the module’s learning content or if this was solely for qualitative analysis. | The prompts of the course learning activities were developed as part of course content to elicit open-ended responses and discussion among participants, and facilitate building knowledge through application of module concepts. We have modified the description for the goal of the course learning activities to make it clear. | p.10-11 |
| 6. For those postdocs who were not on the binary, how were their data treated? Were they dropped for analysis? | We only included those who responded to both the pre- and post-course surveys and excluded the rest for analysis. This has been clarified in our manuscript. We did examine the unmatched data for the purpose of program evaluation, but do not report it in this research manuscript | p.7-8 |
| 7. I know the qualitative results show some trends that URM made more progress than the majority postdocs. Are you able to test for significant improvements? | Thanks for the suggestion. We did find some patterns for URMs with the qualitative data. For example, 76% of URMs perceived mentorship as a contributing factor to achieving the goal they identified, as compared to 62% of majority postdocs. Unfortunately, neither the differences between groups (e.g., URM made more progress than the majority postdocs) nor significant improvement within groups can be tested with the qualitative data. |  |
| Review #2 |  |  |
| 1. This manuscript describes a massive open online course (MOOC) designed to build postdocs’ skills in career transition, career planning, collaborative research, resilience and self-reflection. Quantitative, self-reported learning outcomes data gathered from participants show skills gains in all the targeted areas of learning, with higher gains reported by historically underrepresented participants. Qualitative analysis of work products generated by course participants indicate that networking and mentor support may be crucial to advancing skills in these areas. The premise of this study is thought-provoking, and the results are compelling. I have included some suggestions below to make the article even stronger. | Thanks. We appreciate that the reviewer found our work compelling. |  |
|  |  |  |
| 2.While well-written overall, the article would be strengthened by the inclusion of some additional references and discussion of relevant articles, as space allows. Specifically:• The authors could incorporate more published work on post-doc professional development, and the targeted domains of learning that have been identified and pursued, including work from scholars such as Fuhrmann and Sinche. It was especially odd that the work of Steen et. al was not included since it was referenced in the cover letter as a research article this study was built upon. | Thank you for catching these oversights.. In our revision, we have now incorporated the work of Fuhrmann, Sinche, and Steen that you suggested in the Introduction section.  Sinche M, Layton RL, Brandt PD, O’Connell AB, Hall JD, Freeman AM, Harrell JR, Cook JG, Brennwald PJ. An evidence-based evaluation of transferrable skills and job satisfaction for science PhDs. PloS one. 2017 Sep 20;12(9):e0185023.  Steen K, Vornhagen J, Weinberg ZY, Boulanger-Bertolus J, Rao A, Gardner ME, Subramanian S. A structured professional development curriculum for postdoctoral fellows leads to recognized knowledge growth. PloS one. 2021 Nov 22;16(11):e0260212.  Fuhrmann CN. Enhancing graduate and postdoctoral education to create a sustainable biomedical workforce. Human Gene Therapy. 2016 Nov 1;27(11):871-9. | P1-2 |
| 3.• The authors could include some additional references about the success of using MOOCs as an approach to professional development in general, or specifically for post-doc professional development. | We have added a paragraph discussion using MOOCs as an approach to professional development. We added references addressing the advantages of MOOCs for professional development. We also added three examples of MOOCs as an approach to professional development, such as University Teaching 101, The Inclusive STEM Teaching Project, and Career Discovery Specialization. | p.2 |
| 4. The materials and methods section, data sources, and data analysis sections are well done. This reviewer would suggest a few additions in these sections: • Additional clarification of when and how the participant responses to the six selected prompts are captured. Are these responses collected at a single time point at the end of the course or from a reflection activity which participants can add to over time? | Thank you for the positive comment about our methods and materials section.    The six selected prompts were from the two learning activities: *Mapping Your Goals* and *Putting Your Career Into Action Course*. Learning activities were embedded in the course and learners’ responses were collected and managed through the PostgreSQL database as they progressed through each offering of the course. This has been clarified. More detailed information as for which modules these prompts belong to can be found in S2 Table. | p.10-11 |
| 5. It is noted that 83% of the participants were post-docs; it would help if the authors added information on the career stages of the other 17%, if known. | Thank you for the suggestion. This information has been added to the paragraph describing participants’ characteristics in the Participants part. | p. 8-9 |
| 6. • Additional clarification on how the learners in this study engaged in the various elements and modes of the course. The text notes: “Learners can participate in the course by watching course videos or reading video transcripts, participating in discussions, completing individual reflections, and engaging with interactive learning activities.” It also notes that learners are considered to have completed the course “if they do at least 5 activities.” Are the completed engagement modes and activities tracked? If so, it would be interesting to know if there were any correlations seen between skill gains and levels/types of engagements. | We have added to clarify how the learners in this study engaged in the various elements and modes of the course.  This is a good suggestion and will be considered as one of our future research. However, the current study only examines the differences between the self-perceptions of skills between pre- and post- course surveys and whether they are different as a function of demographic information. | p.4 |
| 7. The quantitative results included in the article are strong and engaging. This reviewer suggests including one additional piece of quantitative data: • Satisfaction/Usefulness of the four modules and the various approaches to learning. It is noted in the discussion: “Also, we believe videos that share stories and perspectives of diverse postdocs invite diverse learners to engage. We know from previous work that integrating self-reflection prompts and interactive activities create opportunities for participants to apply module concepts and directly facilitate their learning.” This discussion point could be supported more strongly with satisfaction data from learners across the content and modalities. | This is a great point. Unfortunately, we don’t have satisfaction data from learners across content and modalities in each module. We only have a proxy for the satisfaction measure (i.e., Would you like to recommend this module to others?).  Our statement here is based on our prior work (Hokanson et al., 2019). We use the literature to support this statement.  Hokanson SC, Grannan S, Greenler R, Gillian-Daniel DL, Campa H, Goldberg BB. A study of synchronous, online professional development workshops for graduate students and postdocs reveals the value of reflection and community building. Innovative Higher Education. 2019 Oct;44(5):385-98. |  |
| 8. The qualitative results included in the article are intriguing and could be expanded and deepened. One possibility includes: • Analyses of the qualitative data by gender and nationality across at least the 4 identified codes. These analyses would strengthen the link between the quantitative and quantitative results. For example, if differences were seen only for URM/non URM learners in both the quantitative and qualitative data, it would enhance understanding. If instead, the only differences seen are in the qualitative data for demographics beyond ethnicity, that would also enhance understanding. Moreover, several points in the discussion related to the qualitative data raise questions about other identities. For example, the literature exploring gender, balancing multiple responsibilities and resilience skills, identity/ belonging and nationality might cause one to hypothesize differences in responses to the qualitative prompts. | Thanks for this suggestion.  We added the analyses of the qualitative data by gender, discipline and country across the 4 codes and displayed the results in S1-S3 Figure. Only one consistent difference was noted by gender across the prompts, which is that the theme of networking was more prominent for women postdocs. For example, 41% of female postdocs perceived lack of skills in networking as a major barrier to career preparation, social connections, or professional development. In contrast, only 19% of male postdocs reported the same challenge. Other than this, no clear patterns were identified in the comparison of codes across different groups. The noted pattern was described in the Results and Discussion sections | p.22 p.24 |
| 9. The discussion raises some interesting points and would benefit from some deeper exploration. For example, the authors state that “Our findings can be interpreted as a readily accessible, pedagogically inclusive and interactive means for URM postdocs to advance their skills, thus reducing the mentoring gap.” Do the authors have evidence that the learners felt the modules filled in a gap in their mentoring? Is it possible that the course did not fill a gap but rather heightened awareness about the need to expand their mentoring network to meet needs outside the course? Another example: the authors state “In the third module, Developing Resilience, we engage postdocs in learning, reflecting on, and applying resilience skills, and provided more information about resources and support services for emotional support or mental and psychological well-being.” Do the authors have data from learners that these resources were useful? | Thanks for the suggestion.  We revised these expressions in the discussion: “Our course helps postdocs heighten the awareness of broadening their mentoring network to meet multifaceted needs for their future success.”  We removed the statement “In the third module, Developing Resilience, we engage postdocs in learning, reflecting on, and applying resilience skills, and provided more information about resources and support services for emotional support or mental and psychological well-being.” | p.25-26 |
| Minor edits: • I believe the reference to the S1 text should be to S2 on page 11 • The identification of the quotes from URM or non-URM should be consistent in the results | It should be S1 text since each type (i.e., text, table, figure) of supplementary material is numbered independently.  The quotes have been made consistent in the results. |  |
